# Sex-Interacting mRNA- and miRNA-eQTLs and Their Implications in Gene Expression Regulation and Disease

**DOI:** 10.3389/fgene.2019.00313

**Published:** 2019-04-09

**Authors:** Jiangshan J. Shen, Yong-Fei Wang, Wanling Yang

**Affiliations:** ^1^Department of Paediatrics and Adolescent Medicine, Li Ka Shing Faculty of Medicine, The University of Hong Kong, Pok Fu Lam, Hong Kong; ^2^Collaborative Innovation Center for Birth Defect Research and Transformation of Shandong Province, Jining Medical University, Jining, China; ^3^Lupus Research Institute, Affiliated Hospital of Jining Medical University, Jining, China

**Keywords:** sexual dimorphism, RNA-Seq, eQTL, microRNA, transcriptome

## Abstract

Despite sex being an important epidemiological and physiological factor, not much is known about how sex works to interact with genotypes to result in different phenotypes. Both messenger RNA (mRNA) and microRNA (miRNA) may be differentially expressed between the sexes in different physiological conditions, and both may be differentially regulated between males and females. Using whole transcriptome data on lymphoblastoid cell lines from 338 samples of European origin, we tried to uncover genes differentially expressed between the two sexes and sex-interacting expression quantitative trait loci (ss-eQTLs). Two miRNAs were found to be differentially expressed between the two sexes, both of which were found to be functionally implicated in breast cancer. Using two stage linear regression analysis, 21 mRNA ss-eQTL and 3 miRNA ss-eQTLs were discovered. We replicated two of the mRNA ss-eQTLs (*p* < 0.1) using a separate dataset of gene expression data derived from monocytes. Three mRNA ss-eQTLs are in high linkage disequilibrium with variants also found to be associated with sexually dimorphic traits. Taken together, we believe the ss-eQTLs presented will assist researchers in uncovering the basis of sex-biased gene expression regulation, and ultimately help us understand the genetic basis of differences in phenotypes between sexes.

## Introduction

Sexual dimorphism is common among different traits and diseases, as exemplified by the differential disease prevalence and severity in autoimmune diseases (Lockshin, 2006). Hormonal milieu, environmental conditions and genetics may all play a role in the sexual dimorphism of autoimmune diseases (Invernizzi et al., 2009). In addition, previous studies have shown that gene by sex interaction effects can be detected in many complex traits as varied as height and life time reproductive success (Rawlik et al., 2016). In mice, both phenotypic presentation after gene knock-outs and wildtype phenotypes were found to differ between males and females in >10% of the 234 traits analyzed (Karp et al., 2017). Although these studies point to there being a genetic effect on sexual dimorphism, the underlying molecular mechanisms are still poorly elucidated. To understand the mechanisms of sexual dimorphism, gene expression is a useful intermediate phenotype that has been shown to be modified by sex (Dimas et al., 2012; Yao et al., 2013; Kukurba et al., 2016), and can be investigated through expression quantitative trait loci (eQTLs).

Expression quantitative trait loci refers to a statistical association between a genotype and gene expression (Dimas et al., 2009), and have been used to pinpoint potential functional variants in the genome and elements that may regulate gene expression. Sex-interacting eQTLs (ss-eQTL) refers to an association between a genotype and gene expression that are statistically different between males and females, and they may shed light on the mechanisms of differential gene regulation between males and females. Many recent studies attempted to uncover ss-eQTLs, and some loci have been found (Dimas et al., 2012; Yao et al., 2013; Kukurba et al., 2016). In Yao, Joehanes (Yao et al., 2013), 14 messenger RNA (mRNA) ss-eQTLs have been found using whole blood-derived RNA-Seq gene expression data from 5254 individuals. All have been discovered to be in high linkage disequilibrium (LD) with variants known to be associated with complex traits in humans (Yao et al., 2014), hinting at the possibility of these SNPs having molecular functional significance in affecting the sexual dimorphism of said complex traits. In a study with 922 Major Depressive Disorder patients and controls, Kukurba, Parsana (Kukurba et al., 2016) uncovered 6 genome-wide ss-eQTLs, two of which is on the X chromosome. This is in contrast to 10915 *cis*-eQTLs uncovered in the same dataset (Battle et al., 2014), suggesting that the majority of *cis*-eQTLs are not sex interacting or the power of detecting sex interaction eQTLs is much lower than the power to discover *cis*-eQTLs.

MicroRNAs (miRNA) are regulatory non-coding RNAs that are 19–23 nucleotides in length and they partially bind to complementary transcripts that are the targets of their regulatory action. Like other transcripts, miRNA may also have differential gene expression between the sexes, and may also be regulated differently between the sexes (Sharma and Eghbali, 2014). Interestingly, sex hormone regulated miRNA have been implicated in diseases ranging from psychiatric (Mellios et al., 2010) to autoimmune and metabolic diseases (Wang et al., 2012). Although some studies have attempted to find the elements regulating miRNAs through microRNA eQTLs (miR-eQTLs) (Gamazon et al., 2013), to date no study has attempted to find sex-interacting microRNA eQTLs (miRNA ss-eQTLs). To contribute to this emerging field, we use data from the thoroughly sequenced Geuvadis consortium, which consists of whole transcriptome and SNP genotype data from 338 European samples, to look for sex-interacting eQTLs (ss-eQTLs) in both mRNA and miRNAs. We also use as a replication cohort of stimulated immune cells gene expression data from 367 individuals to attempt to replicate the mRNA ss-eQTLs (Fairfax et al., 2014).

The X chromosome differs in the number of copies between males and females and may be expected to play an important role in sex-biased gene regulation. Interestingly, an enrichment of ss-eQTLs have been found on the X chromosome (Yao et al., 2013; Kukurba et al., 2016), though the effect sizes of ss-eQTLs on the X chromosome tend to be smaller (Kukurba et al., 2016). Furthermore, when compared to the autosomes, Kukurba, Parsana (Kukurba et al., 2016) found an enrichment of open chromatin regions that are sex specific on the X chromosome. They also found an enrichment of genes with sex-biased gene expression in sex-specific open chromatin, as well as an enrichment of ss-eQTL in such open chromatins, suggesting molecular mechanisms underlying sex specific gene regulation may be detected through these genomic approaches (Kukurba et al., 2016). In our study, we also investigated the presence and enrichment of ss-eQTLs on the X chromosome. Together, the autosomal and X chromosome based ss-eQTLs may have implication for differential gene regulation between sexes, as well as differential disease prevalence and severity between sexes.

## Materials and Methods

### Sample Description

With the overarching aim of discovering ss-eQTLs, we analyzed data from the Geuvadis consortium (Lappalainen et al., 2013). The Geuvadis consortium generated both RNA-Seq and miRNA seq data in lymphoblastoid cell lines (Lappalainen et al., 2013). Gene expression and SNP genotype data across 338 European samples consisting of 162 male and 176 females was used to uncover mRNA ss-eQTL. MiRNA expression and SNP genotype data from 155 males and 171 females was used to uncover sex-interacting miRNA eQTLs (miRNA ss-eQTL). We refer to both miRNA and mRNA sex-interacting eQTL together as ss-eQTLs. Genotypes were downloaded from 1000 genome project phase 3 for the genotypes corresponding to the gene expression samples (The 1000 Genomes Project Consortium, 2015). To limit our search to cis acting ss-eQTLs, we filtered for genotypes that are within 1MB of the transcription start site (TSS) of genes or within 1MB of miRNAs. Only biallelic SNPs and SNPs above a minor allele frequency (MAF) of 1% were used in our study.

### Two Stage Regression for Uncovering Sex-Interacting eQTLs

To locate ss-eQTLs, we applied the following linear regression equation:

(1) $$y=\beta_{0}+\beta_{1}SNP+\beta_{2}sex+\beta_{3}sex*SNP+\beta_{4}PC1+\beta_{5}PC2+\beta_{6}PC3+e$$

where y is the normalized gene expression value downloaded from^1^ in December 2014, SNP is coded as 0, 1, 2 for the dosage of alternative alleles, and sex is coded as 0, 1 for male and female, respectively. The sex^∗^SNP term refers to the interaction term between sex and genotype. PC1, PC2, and PC3 refer to the principal components (PCs) reflecting population stratification, as calculated from Eigenstrat (Price et al., 2006). As Lappalainen et al. (2013) have shown, the first three PCs are enough to correct for population stratification in the Geuvadis dataset. As per usual linear regression norms, e refers to the error term and the β’s are the coefficients of the linear regression. As the downloaded gene expression values were already normalized for linear regression analysis, and batch effects along with other confounding variables were removed, we did not do further normalization. Four individual samples, two males and two females, were discarded as outliers after the Eigenstrat analysis, possibly due to cryptic relatedness. This resulted in a total sample size of 174 males and 160 females for the mRNA ss-eQTL tests and 155 males and 171 females for miRNA ss-eQTL tests.

In total, 3,913,830 SNP/miRNA combinations and 515,683,907 SNP/mRNA combinations were tested, comprised of 715 miRNA and 23722 mRNA transcripts, respectively. The Benjamini-Hochberg false discovery rate (FDR) thresholds for mRNA and miRNA analyses were calculated separately. Potentially statistically significant eQTLs were defined as ones that fell below the FDR threshold of 0.05 for β_3_, equivalent to the *P*-value threshold of 1.364019e-05 for mRNA and 2.052497e-05 for miRNA, respectively.

To minimize false positives, a second stage of regression analyses were performed on ss-eQTLs that are potentially significant, according to the *P*-value cutoff from stage one. The samples were separated into male samples only and female samples only, and a separate regression analysis was performed on each subset of samples. Linear regression estimates from least square method are based on the assumption that the error term is normally distributed, and deviation from that assumption may lead to estimates that are inaccurate (Ruckstuhl, 2014). To ameliorate influence from outliers, we performed this second round of linear regression using a robust fit of regression model (RLM) containing an M estimator using the following equation:

(2) $$y=\beta_{0}+\beta_{1}SNP+\beta_{2}PC1+\beta_{3}PC2+\beta_{4}PC3+e$$

As in equation (1), y is the normalized gene expression value, SNP refers genotype coded as dosage of alternative alleles and PC1, PC2 and PC3 refer to the PCs delineating population structure. Equation (2) is fitted separately for male and female samples. To perform the fitting, the rlm() function in R (version 3.3.2) was used with default settings and fitting was done by iterated re-weighted least squares (IWLS). To evaluate the significance of coefficients in the robust linear model (RLM), f.robtest() function in R was used, which encodes a Wald’s test that robustly tests coefficients of an RLM.

After equation (2), we applied the following filter for quality control:

(1) All genotype by sex blocks must have at least 5 data points and all three genotypes (0, 1, 2) must be present.
(2) β_1_ from equation (2) must pass the stage 2 FDR 0.05 thresholds: *p* < 0.02635554 for mRNA and *p* < 0.01305914 for miRNA. If the p for β_1_ in either male samples, female samples, or both pass the p cutoff in a single tested ss-eQTL, we called that ss-eQTL significant.

On the X chromosome, we tested SNP/gene expression combinations using the same method above, but with a different encoding for genotypes. We used the encoding 0, 2 for male SNP genotypes and 0, 1, 2 for female SNP genotypes as this is a method that has been used on X chromosome association studies (Gao et al., 2015) and reflects the hemizyous nature of the X chromosome in males, while taking into account X inactivation in females.

### Differentially Expressed miRNA Between Sexes

To search for differentially expressed miRNA between males and females in the European (CEU) population, we analyzed the miRNA expression data using the R package TweeDESeq. TweeDESeq fits the RNA-Seq count data to a family of flexible distributions that can accommodate a variety of shapes of count distributions, such as tail heavy, Poisson and negative binomial. This package takes advantage of the increased sample size to estimate two parameters of count distribution using maximum likelihood. Benjamini-Hochberg (FDR) adjusted *P*-value of 0.05 was used as a cutoff, where miRNA with adjusted P values below the cutoff were determined as having sex-biased expression. Differential mRNA expression analyses for this dataset were previously performed in a similar manner and published elsewhere (Shen et al., 2017).

### Replication Study

For replication of mRNA ss-eQTLs, we used data from Fairfax, Humburg (Fairfax et al., 2014), which contains data from CD14+ monocytes from healthy European volunteers that has been exposed to IFN-γ for 2 h. Illumina HumanHT-12 version 4 Beadchip with 47, 321 gene expression probes was used for assaying gene expression. Genotype data was assayed on Illumina OmniExpress v1.0 chip and downloaded from European Genome Phenome archive (Lappalainen et al., 2015) (accession numbers: EGAD00010000144 and EGAD00010000520). To impute genotype data, we conducted pre-phasing using SHAPEIT (Delaneau et al., 2008). We then conducted imputation using IMPUTE2 (Howie et al., 2009) using 1000 Genomes Project data as reference (Phase I integrated set March 2012 build 37). SNPs with impute INFO score <0.9 were filtered out. SNPs with >5% missing data or with minor allele frequency <1%, and subjects with >5% missing data were removed. We then tested for Hardy-Weinberg equilibrium (HWE) in each GWAS dataset and removed SNPs with HWE *p* < 1e10^-4^. Sex of all 367 samples was imputed from the genotype data using PLINK (Purcell et al., 2007). Only the 21 significant mRNA ss-eQTLs were checked to see if they were also ss-eQTLs in the replication dataset.

### Annotation of Sex-Interacting eQTLs

To annotate the ss-eQTLs, we looked for co-localization between the ss-eQTLs and the GRASP catalog (Leslie et al., 2014), which catalogs genotype-phenotype studies from 1390 GWA studies. We investigated ss-eQTLs which are in high LD (*R*^2^ > 0.8) with SNPs which have nominal associations with a phenotype under the condition of *p* < 0.05. We also annotated the genes and miRNAs that are regulated by ss-eQTLs through literature searches. The workflow of the study can be found in a flowchart in Supplementary Figure 1.

## Results

Genes can be differentially expressed between males and females. Using the same dataset, we previously found 587 differentially expressed mRNAs and have uncovered two differentially expressed miRNA between males and females (Shen et al., 2017; Table 1). Using a lenient cutoff of *p* < 0.1, 2 out of the 21 mRNA ss-eQTL were replicated (Table 2). No X chromosome ss-eQTLs were found.

**Table 1:** Differentially expressed miRNA between males and females in European samples.

Chromosome	GRCH 37 coordinates	Target_ID	Mean expression male	Mean expression female	Fold change in gene expression	Adjusted *P*-value
X	7065910	hsa-miR-4767-5p	8.37	16.03	1.92	2.18E-05
X	8095021	hsa-miR-651-5p	11.93	19.20	1.60	0.02872


**Table 2:** Significant sex interacting eQTLs of mRNA expression (mRNA ss-eQTLs).

RSID	Gene name	Female coefficients	Male coefficients	Female *P*-value	Male *P*-value	Replication *P*-value
rs112326775	GDAP2	0.339539627	-0.11718	2.05E-06	0.143830519	
rs112974903	AIM2	-1.868517737	1.106382	9.78E-05	0.02635554	
rs11590749	SLAMF6	-3.379121873	1.841065	0.000344	0.019310424	
rs12565300	RLF	0.26324412	-0.3431	0.011447	0.002140632	
rs75248030	ATG4C	-0.334882669	0.508386	0.014263	0.00397029	
rs149151379	TMEM218	-1.399994261	1.002162	0.000228	0.015328711	0.0624
rs16929747	C11orf74	0.36683671	-0.2813	0.000602	0.035350972	0.91
rs4766961	RAB35	1.496733959	-2.7356	0.022816	7.12E-05	0.82
rs513203	TMEM5	0.466920531	-0.41312	0.004639	0.025651151	0.56
rs11054441	CDCA3	1.992907146	-3.01645	0.009764	0.000511027	0.35
rs117057667	ERCC5	0.76003468	-2.3343	0.137681	6.09E-06	
rs4769750	GJB6	-0.016458075	0.010116	0.007125	0.071033232	0.288
rs112147266	SNTB2	-0.10862917	1.096127	0.595559	1.77E-06	0.45
rs71368142	SPNS3	-0.087973185	0.070989	0.001521	0.009632486	0.394
rs11078646	XAF1	-7.066113461	2.396446	9.12E-07	0.109016282	0.67
rs72875017	RBBP8	0.408844502	-0.50581	0.004991	0.002459293	
rs3791929	RUFY4	0.164720607	-0.57524	0.094027	7.83E-07	
rs7846370	CA2	-0.531896699	0.747268	0.010103	0.001137671	0.80
rs17147185	RAPGEF1	-1.69609387	1.341208	0.002645	0.021712081	
rs4442263	FUT7	-0.380740056	0.707437	0.050054	0.000827635	0.94
rs72618132	HNRNPK	-10.54774468	11.44877	0.000618	0.000378394	0.095


*Female coefficients refers to β_*1*_ in equation 2, when only female samples were used. Female *P*-value refers to the *P*-values associated with β_*1*_. Male coefficients refers to β_*1*_ in equation 2, when only male samples were used. Male *P*-value refers to the *P*-values associated with β_*1*_. Replication *P*-value refers to p values associated β_*3*_ of equation (1) in the replication cohort. Empty replication *P*-value cells are due to the data not being present in the replication cohort.*

**FIGURE 1:**
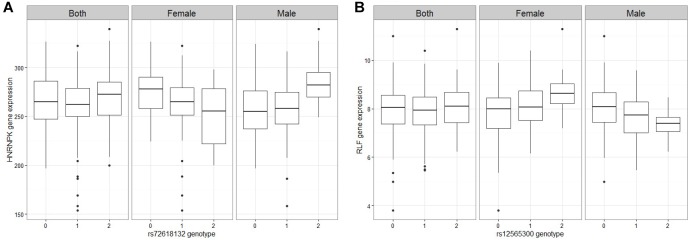
Figures of representative mRNA ss-eQTLs. Genotypes are labeled based on the whether they are homozygote reference/reference (0), ref/alt (1), or alt/alt (2). In each figure, we plot the gene expression values against the genotype when both male and female samples are used, and when only female samples are used and when only male samples are used. **(A)** HNRNPK against rs72618132 **(B)** RLF against rs12565300.

**FIGURE 2:**
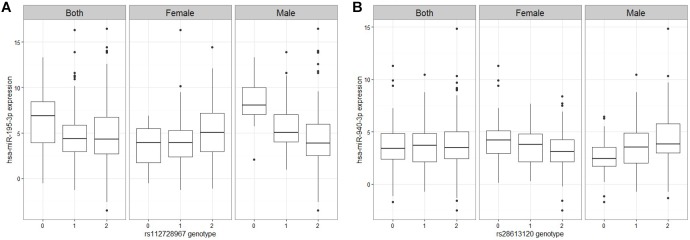
Figures of representative miRNA ss-eQTLs. Genotypes are labeled based on the whether they are homozygote reference/reference (0), ref/alt (1), or alt/alt (2). In each figure, we plot the miRNA expression values against the genotype when both male and female samples are used, and when only female samples are used and when only male samples are used. **(A)** hsa-miR-195-3p against rs112728967 **(B)** hsa-miR-940-3p against rs28613120.

A linear regression of gene expression values against the genotype and genotype by sex interaction term was used to uncover ss-eQTLs. This was followed by a regression analysis where males and females were separately evaluated for whether the potential ss-eQTLs contained genotypes that were statistically associated with gene expression. Using two stage regression analysis, we uncovered 21 mRNA ss-eQTL (Table 2 and Figure 1A,B) and 3 miRNA ss-eQTLs (Table 3 and Figure 2A,B) at minor allele frequency (MAF) cutoff >0.01.

**Table 3:** Significant sex interacting eQTLs of microRNA expression (miRNA ss-eQTLs).

RSID	Chromosome	Genotype coefficient	Genotype *P*-value	Interaction coefficient	Interaction *P*-value	microRNA
rs28613120	chr16	0.992057	0.000275	-1.6696	9.06E-06	hsa-miR-940-3p
rs112728967	chr17	-1.64553	6.82E-05	2.545309	1.63E-05	hsa-miR-195-3p
rs4956019	chr4	0.843895	0.011082	-2.02319	1.89E-05	hsa-miR-576-3p


*Genotype coefficient refers β_*3*_ in equation 1, Genotype *P*-value refers to the ps associated with β_*1*_. Interaction coefficient refers to β_*3*_, and Interaction *P*-value refers to the ps associated with β_*3*_.*

To annotate ss-eQTLs, co-localization ANALYSIS of the ss-eQTLs and variants that are associated with complex traits was also performed. Three ss-eQTLs, both mRNA and miRNA, were in high LD with at least one other variant in the GRASP database, and displayed interesting patterns in the phenotypes these ss-eQTLs might be involved in (Table 4).

**Table 4:** Annotation of ss-eQTLs with entries from the GRASP database.

RSID of ss-eQTL	Gene name of ss-eQTL	GRASP *P*-value	GRASP trait	GRASP SNP RSID
rs4769750	GJB6	0.010885	Advanced age related macular degeneration	rs1994539
rs4766961	RAB35	0.003954	HDL cholesterol change with statins	rs4766961
rs4769750	GJB6	0.014932	HDL cholesterol change with statins	rs1994539
rs4766961	RAB35	0.0069	HDL cholesterol change with statins	rs4766962
rs7846370	CA2	0.009	Albuminuria	rs1483767
rs4766961	RAB35	0.0342	Rheumatoid Arthritis	rs4766961
rs4766961	RAB35	1.3E-09	Gene expression of RNF10	rs4766961
rs4766961	RAB35	1.8E-10	Gene expression of COQ5	rs4766961


*All entries listed have *R*^*2*^ > 0.8 with the ss-eQTLs, and the GRASP *P*-value and trait are also listed.*

## Discussion

Similar to results from previous studies (Dimas et al., 2012; Kukurba et al., 2016), none of the target mRNA and miRNAs of ss-eQTLs were significantly differentially expressed between males and females. Neither mRNA nor miRNA ss-eQTLs correspond to original *cis*-eQTLs found using the same dataset (Lappalainen et al., 2013), suggesting that previous *cis*-eQTLs were not differentially regulated by sex.

Interestingly, no X chromosome ss-eQTLs were found, in contrast to Kukurba et al.’s (2016) finding that ss-eQTLs are more likely to be on the X chromosome. Traditionally, power to detect eQTLs are lower on the X chromosome due to the lower quality of genotypes assayed on the X chromosome (Gao et al., 2015), and due to the escaping of X inactivation leading to more variation in gene expression between sexes (Carrel and Willard, 2005). In addition, Kukurba et al. (2016) have found ss-eQTLs on the X chromosome have lower effect sizes on average than ss-eQTLs on the autosomes, making it harder to detect ss-eQTL on the X chromosome. Therefore, it’s possible our current samples do not have enough power to detect ss-eQTL on the X chromosome. The two differentially expressed miRNA were both located on the X chromosome, possibly due to there being a higher density of miRNA on the X chromosome compared to the Y chromosome (Pinheiro et al., 2011). Some of the X-linked miRNA may escape X inactivation, which may lead to a higher likelihood of sex biased miRNAs on the X chromosome. In fact, both of the sex biased miRNA are in regions of the X chromosome where escape genes are sometimes found (Carrel and Willard, 2005), and it may be that they are differentially expressed between males and females due to the escaping of X inactivation. Interestingly, both of the sex-biased miRNAs were functionally implicated in breast cancer: miR-4767 was first identified in a breast cancer cell line MCF7 (Persson et al., 2011) and hsa-miR-651-5p was found to be one of the miRNAs whose increased expression predicted increased breast cancer survival (Chang et al., 2016).

### Replication of Sex-Interacting eQTLs and Study Limitations

Detection and replication of ss-eQTLs may be limited by the increased sample size required to detect sex-interacting effects, the tissue specificity of sex-biased gene expression and regulation, and the different statistical methods used across studies. Only a few studies have been conducted in recent years to investigate sex-interacting eQTLs. Dimas, Nica (Dimas et al., 2012) uncovered 109 threshold based sex discordant eQTLs in HapMap CEU population despite a small sample size of only 54 females and 55 males. The Framingham study on the other hand, uncovered only 14 sex interacting eQTLs in a sample of 2833 female and 2421 males (Yao et al., 2013). Most recently, Kukurba et al. (2016) uncovered 6 sex interacting eQTLs genome wide, using 922 whole blood, RNA-Seq samples. Two of the genome-wide significant ss-eQTLs replicated between the Kurkuba and the Framingham and Cartagene study. However, many of the eQTLs do not replicate across studies this points to the difficulty in replicating ss-eQTLs in different cohorts, perhaps due to both methodological differences and biological sample differences. Dimas et al. (2012) used RNA-Seq data from lymphoblastoid cell-line and a threshold based method adapted from Storey (2002), whereas the Framingham study used whole blood transcriptome data and linear regression followed by permutation (Yao et al., 2014). The Kukurba et al. (2016) study used methods similar to (Yao et al., 2014), but used the whole blood transcriptome data from Major Depressive Disorder patients in addition to healthy controls. Sexual dimorphism in gene expression is known to have tissue specificity (Gershoni and Pietrokovski, 2017), and that may also apply to sex specific gene regulation. As the different studies mentioned above all used different tissues under different biological conditions, it may account for some lack of replicability across studies. In addition, the large number of sample sizes required to uncover sex interacting eQTLs may play a role: Leon and Heo (2009) estimated that it takes four times the number of samples required to uncover an eQTL to uncover a sex-interaction effect.

In order to better understand the power to detect ss-eQTLs, we performed a power analysis. We first used the G^∗^Power software (Ruckstuhl, 2014) to calculate the power for the interaction term under the assumption of an ANOVA test without any covariates. The actual power would be lower as we used a multivariate regression, and this is essentially testing a univariate regression with only the interaction term. This analysis showed that assuming a small effect size of 0.1, the power of this study is 0.24, but this goes up if the effect size is larger. For example, at the effect size of 0.2, the power is 0.81. Supplementary Figure 2 shows the power as a function of effect size. We then attempted to perform a power analysis of the second stage of the regression using the R power powerEQTL. As can be seen from the figure below, the power to detect an eQTL depends on the MAF of the genotype and the sample size. For the sample size of 176 for females and the sample of 162 males, there is good power to detect an eQTL if MAF is above 0.15. Our average MAF for the mRNA ss-eQTL is 0.19, ranging from 0.036 to 0.42, suggesting that if the first stage regression worked well, then there is good power to detect eQTL in the second stage. Supplementary Figure 3 shows the change in power as a function of the MAF and sample size.

### Annotation of Sex-Interacting eQTLs

Co-localization of the ss-eQTLs and variants that are associated with complex traits may suggest a functional role of the ss-eQTL locus; such a co-localization of the ss-eQTLs was investigated through using the Genome-Wide Repository of Associations Between SNPs and Phenotypes (GRASP) database (Leslie et al., 2014). The (GRASP) database contains genome wide association studies of variants and phenotypes with nominal association *p* < 0.05 Gene expression in whole blood were the most significant variants the ss-eQTLs were in LD with, confirming that some of the ss-eQTLs we found were also eQTLs in other studies. Interestingly, variants associated with rheumatoid arthritis, an autoimmune disease with sex differences in prevalence (Weyand et al., 1998), were also found to be in high LD with one of the ss-eQTLs. Also interestingly, 2 ss-eQTLs were in high LD with variants associated with HDL cholesterol response to statin, another phenotype with known sex differences (Karp et al., 2007). As well, albuminuria, the presence of albumin in urine, a possibly sexually dimorphic trait (Nitsch et al., 2013), was also found to be in high LD with ss-eQTLs. Advanced age related macular degeneration, a disease with known sex specific associated genetic variants, were found in high LD with a ss-eQTLs as well (Sasaki et al., 2018). Results are displayed in Table 4.

Of the 2 ss-eQTLs that were replicated, one of the target gene, *Hnrnpk*, has been linked to the neuroendocrine differentiation signaling in prostate cancer (Ciarlo et al., 2012), to the development of breast cancer (Hamrita et al., 2008), and has been suggested to be regulated by sex hormones (Ciarlo et al., 2012), providing some evidence to support its differential regulation between sexes. Overall, there is some evidence to suggest that the ss-eQTLs show an association to sexually dimorphic traits and may help us elucidate the molecular mechanisms behind such dimorphism.

This study is the first study to our knowledge that investigated sex-interacting eQTLs in miRNA, and the first study to use two stage regression to investigate ss-eQTLs. Although recent studies suggest that there exist genetic contributions to sexual dimorphisms in complex traits (Rawlik et al., 2016), at the gene expression level, we may not detect many sex-interacting eQTLs in a specific tissue. Increased sample sizes, and increased studies in more tissues may uncover more ss-eQTLs and increase our understanding of sexual dimorphism in gene regulation.

## Author Contributions

JS and WY contributed to conception and design of the study. JS performed the statistical analysis. All authors contributed to manuscript revision and read and approved the submitted version.

## Conflict of Interest Statement

The authors declare that the research was conducted in the absence of any commercial or financial relationships that could be construed as a potential conflict of interest.

## Footnotes

1 ftp://ftp.ebi.ac.uk/pub/databases/microarray/data/experiment/GEUV/E-GEUV-1/analysis_results/
