# Supplementary material for: Sex-Interacting mRNA- and miRNA-eQTLs and Their Implications in Gene Expression Regulation and Disease
Source: Front Genet. 2019 Apr 9;10:313. doi: 10.3389/fgene.2019.00313 (PMC6465513; doi:10.3389/fgene.2019.00313)
Supplement: Supplementary file 1 [file Image_1.pdf]

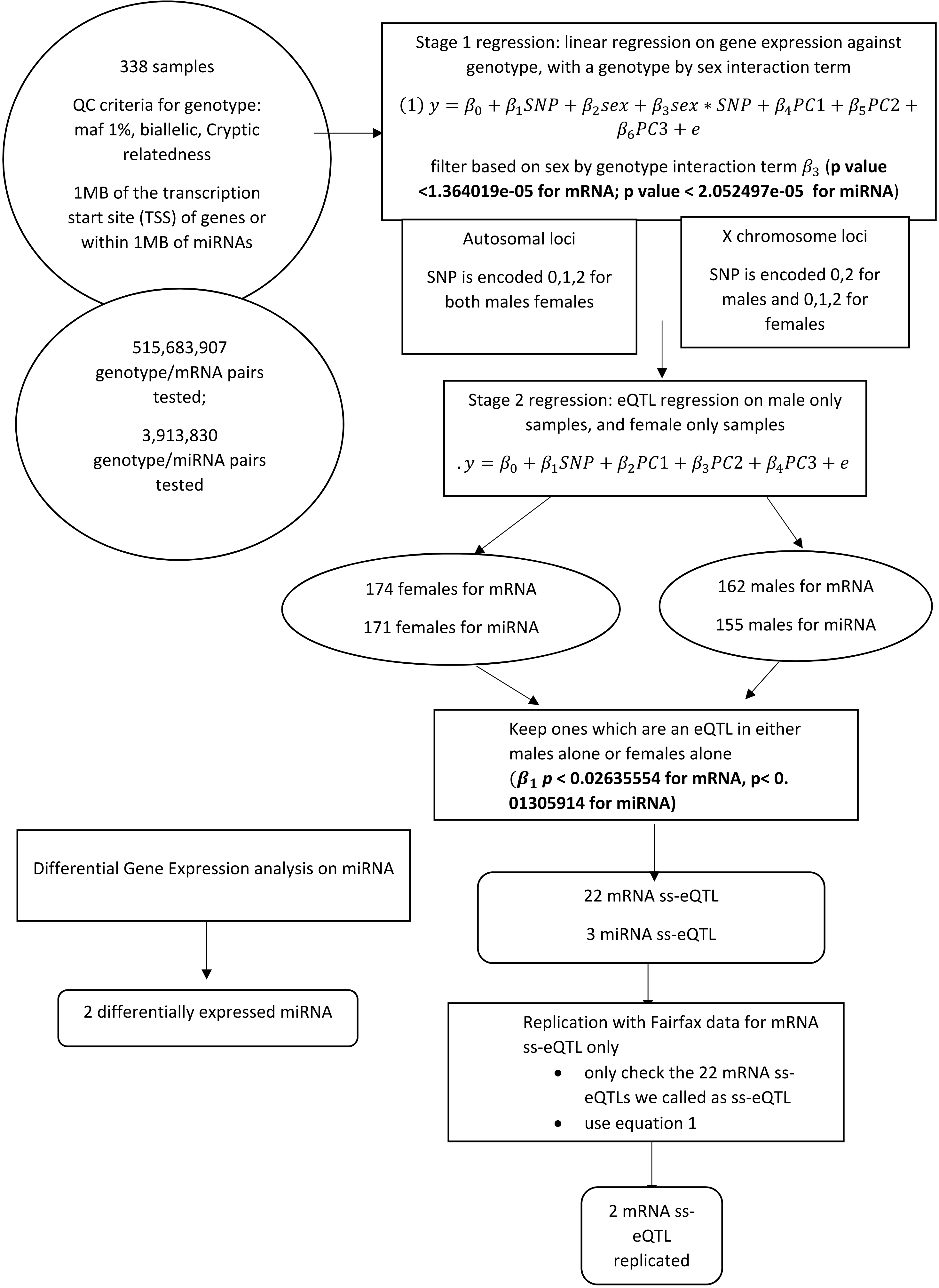
338 samples
QC criteria for genotype: maf 1%, biallelic, Cryptic relatedness
1MB of the transcription start site (TSS) of genes or within 1MB of miRNAs
515,683,907 genotype/mRNA pairs tested;
3,913,830 genotype/miRNA pairs tested
Stage 1 regression: linear regression on gene expression against genotype, with a genotype by sex interaction term
$(1)\ y = \beta_0 + \beta_1 SNP + \beta_2 sex + \beta_3 sex * SNP + \beta_4 PC1 + \beta_5 PC2 + \beta_6 PC3 + e$
filter based on sex by genotype interaction term β_3 (**p value <1.364019e-05 for mRNA; p value < 2.052497e-05 for miRNA**)
Autosomal loci
SNP is encoded 0,1,2 for both males females
X chromosome loci
SNP is encoded 0,2 for males and 0,1,2 for females
Stage 2 regression: eQTL regression on male only samples, and female only samples
$. y = \beta_0 + \beta_1 SNP + \beta_2 PC1 + \beta_3 PC2 + \beta_4 PC3 + e$
174 females for mRNA
171 females for miRNA
162 males for mRNA
155 males for miRNA
Keep ones which are an eQTL in either males alone or females alone
(**β_1 p < 0.02635554 for mRNA, p< 0. 01305914 for miRNA)**
Differential Gene Expression analysis on miRNA
2 differentially expressed miRNA
22 mRNA ss-eQTL
3 miRNA ss-eQTL
Replication with Fairfax data for mRNA ss-eQTL only
• only check the 22 mRNA ss-eQTLs we called as ss-eQTL
• use equation 1
2 mRNA ss-eQTL replicated

Supplementary Figure 1: Flowchart showing the steps taken in analysis of ss-eQTLs, from quality control, to 2 stage regression analysis, to replication.
