# Supplementary material for: Sex-Interacting mRNA- and miRNA-eQTLs and Their Implications in Gene Expression Regulation and Disease
Source: Front Genet. 2019 Apr 9;10:313. doi: 10.3389/fgene.2019.00313 (PMC6465513; doi:10.3389/fgene.2019.00313)
Supplement: Supplementary file 2 [file Image_2.pdf]

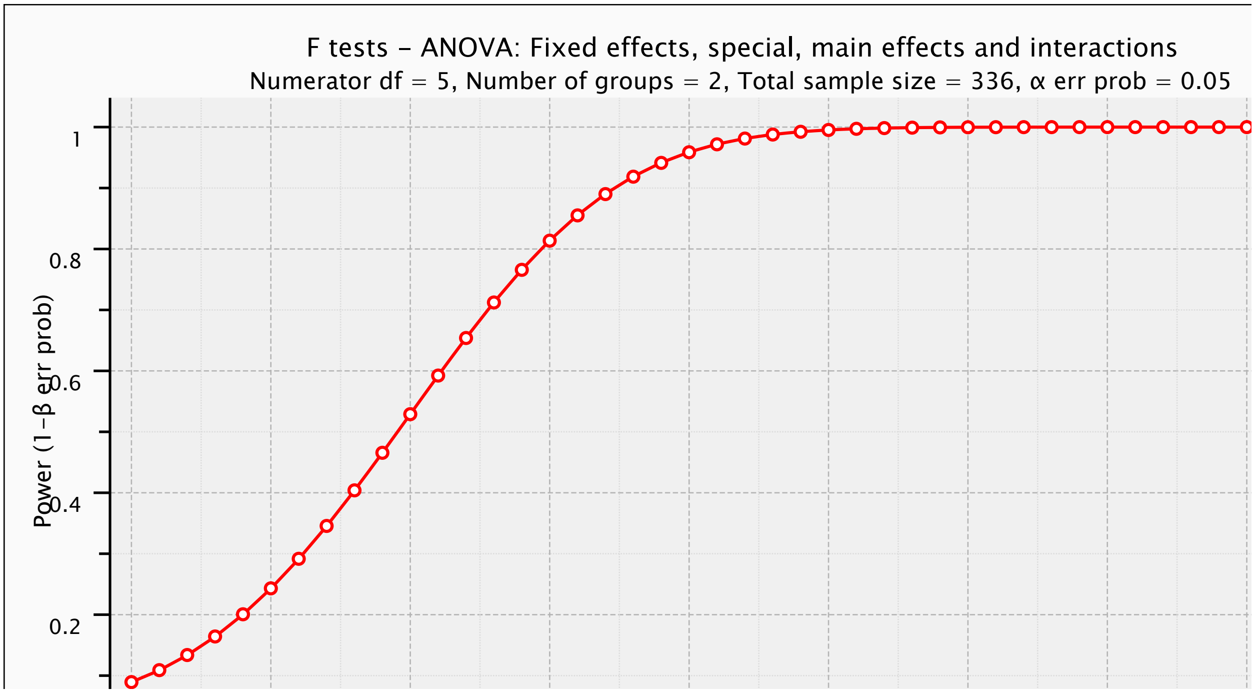

Supplementary Figure 2: The power of doing an eQTL analysis using ANOVA with an interaction term, calculated using G*POWER. This is a post hoc analysis of the power at various effect sizes, with a sample size of 336, type I error = 0.05, and 2 groups (male and female). The significance level is set at 0.05, and Number of groups (male vs female) set at 2.
