# Supplementary material for: Sex-Interacting mRNA- and miRNA-eQTLs and Their Implications in Gene Expression Regulation and Disease
Source: Front Genet. 2019 Apr 9;10:313. doi: 10.3389/fgene.2019.00313 (PMC6465513; doi:10.3389/fgene.2019.00313)
Supplement: Supplementary file 3 [file Image_3.pdf]

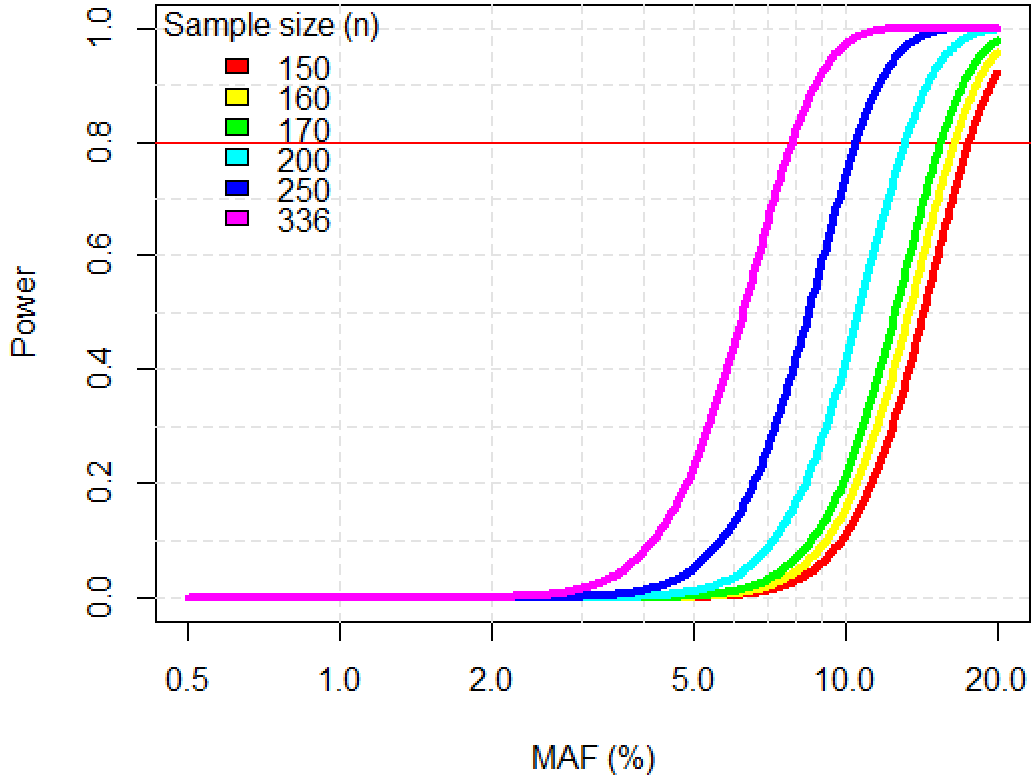

Supplementary Figure 3: Power analysis for eQTL using simple linear regression, such as the analysis we performed in stage 2 of the 2 stage regression analysis. The analysis was done using the powerEQTL R package. This plot shows the power of the study as a function of different minor allele frequencies (MAF) .
